# Severe sepsis with multiple organ dysfunctions caused by *Pseudomonas aeruginosa *in an immunocompetent child

**DOI:** 10.1186/cc11756

**Published:** 2012-11-14

**Authors:** G Jugulete, M Luminos, A Visan, A Draganescu, M Merisescu, M Vasile, A Nica

**Affiliations:** 1 Institute of Infectious Diseases, Bucharest, Romania

## Introduction

*Pseudomonas aeruginosa *is a Gram-negative bacillus, which can cause severe infections especially in immunocompromised patients. Most infections caused by this germ are nosocomial because of increased antibiotic and antiseptic resistance but in rare cases we talk about community infections. Severe infection in immunocompetent patients caused by this microbe is extremely rare. The mortality rate in sepsis caused by *P. aeruginosa *is very high (up to 60%).

## Methods

We present the case of a 2-year-old boy admitted to our clinic for fever, diarrhea, impaired general condition, vesiculobullous and petechial exanthema. From the child's medical history we would like to mention: untreated perianal fissure and excised warts on terminal phalanges of his third finger of the left hand, big toe and the fifth toe of his left leg as the overgrowth and necrosis at the site of excision. Shortly after admission the patient presents an episode of acute respiratory failure followed by apnea and he is tracheal intubated and mechanically ventilated.

## Results

During admission the general condition remains serious, the patient remains sedated and respiratory assisted with petechial exanthema, with hypotension, oligo-anuria, anal fissure, and necrotic lesions in the distal finger phalanges. Biological: severe pancytopenia (WBC = 700/mmc, Hb = 8.2 g/dl, platelets = 34.000/mmc), coagulation disorders, nitrogen retention syndrome, hypoalbuminemia, hepatic cytolysis syndrome, procalcitonin and inflammatory tests intensely positive. On smears made from the skin lesions, Gram-negative bacilli were visualized. The cultures of skin lesions were suggestive for *P. aeruginosa*, confirmed after 48 hours by blood culture identification and PCR determination (Plex-ID). We established comprehensive treatment: antibiotic (meropenem, ciprofloxacin, linezolid), antifungal, inotropic drugs (dobutamine, norepinephrine), ENP (rebalance acid-base and electrolyte), human immunoglobulin, blood and blood products transfusions and Neupogen. The evolution was favorable to the resumption of diuresis after 24 hours, increasing white blood cells and the lesions diminished.

## Conclusion

Positive diagnosis of severe sepsis was based on clinical and laboratory findings. We have established the likely starting point of the patient's severe condition to be the multiple skin lesions and untreated anal fissure. Although the patient was immunocompetent, he developed a severe form of sepsis with septic shock caused by *P. aeruginosa *hardly responsive to treatment. We believe that the patient's favorable development was due to this germ's increased sensitivity to antibiotics, most probably community acquired.

